# Soluble platelet endothelial cell adhesion molecule 1 (sPECAM-1) improves diagnostic accuracy of D-Dimer in patients with suspected deep vein thrombosis (DVT)

**DOI:** 10.1007/s11239-020-02087-7

**Published:** 2020-03-26

**Authors:** Joerg Kellermair, Alexander Fellner, Alexander Bittinger, Stephanie Schneider, Kaveh Akbari, Juergen Kammler, Thomas Lambert, Clemens Steinwender

**Affiliations:** 1 Department of Cardiology, Kepler University Hospital, Medical Faculty Johannes Kepler University Linz, Linz, Austria; 2 Department of Radiology, Kepler University Hospital, Medical Faculty Johannes Kepler University Linz, Linz, Austria; 3 Paracelsus Medical University Salzburg, Salzburg, Austria

**Keywords:** D-dimer, sPECAM-1, Deep vein thrombosis, Biomarker

## Abstract

D-Dimer has a high sensitivity but a low specificity for the diagnosis of deep vein thrombosis (DVT) which limits its implementation as a general screening parameter. There is a demand for additional biomarkers to improve its diagnostic accuracy. Soluble platelet endothelial cell adhesion molecule 1 (sPECAM-1) is generated at the site of venous thrombosis, thus, represents a promising biomarker. Patients with clinically suspected DVT (N = 159) were prospectively recruited and underwent manual compression ultrasonography (CCUS) to confirm or exclude DVT. The diagnostic value of D-Dimer, sPECAM-1 and the combination of both was assessed. sPECAM-1 levels were significantly higher in patients with DVT (N = 44) compared to patients without DVT (N = 115) (85.9 [76.1/98.0] ng/mL versus 68.0 [50.1/86.0] ng/mL; p < 0.001) with a diagnostic sensitivity of 100% and a specificity of 28.7% at the cut point > 50.2 ng/mL. sPECAM-1 improved the diagnostic accuracy of D-Dimer: the combination of both biomarkers yielded a ROC-AUC of 0.925 compared to 0.905 for D-Dimer alone and 0.721 for sPECAM-1 alone with a reduction of false-positive D-Dimer cases 72- > 43 (Δ =  − 31.9%). The discrimination mainly occurred in a subgroup of patients characterized by an inflammatory background (defined by c-reactive protein level > 1 mg/mL). sPECAM-1 represents a novel diagnostic biomarker for venous thrombosis. It does not qualify as a diagnostic biomarker alone but improves the diagnostic accuracy of D-Dimer in patients with suspected DVT.

## Highlights

- sPECAM-1 plasma levels are significantly elevated in patients with acute deep vein thrombosis.
- sPECAM-1 improves the diagnostic accuracy of D-Dimer in patients with suspected acute deep vein thrombosis.
- sPECAM-1 represents a novel diagnostic biomarker for venous thrombosis.

## Background

Venous thromboembolism (VTE; i.e. deep vein thrombosis [DVT] and pulmonary embolism [PE]) is a common disorder with significant socioeconomic consequences due to high morbidity and treatment costs. Diagnosis of DVT is based on imaging techniques including compression ultrasonography (CCUS). In patients with clinically suspected DVT, clinical scoring tools such as the Wells Score [1] are frequently used to predict a priori DVT probability, and to determine if further diagnostic testing is warranted [2]. In patients with low-pretest probability plasma D-Dimer measurement is the first evaluating step as negative test results rule out venous thrombosis [3]. D-Dimer has a high sensitivity [4–6], however, specificity is only about 40–50% [7] and false positive results due to various co-morbidities (i.e. trauma, surgery [8], pregnancy [9], inflammation [10], malignancy) limit its implementation as a general screening test for suspected DVT. In particular, D-Dimer is frequently elevated in patients with an inflammatory background (i.e. infectious diseases [11], sepsis [12], rheumatic diseases [13]) characterized by elevated unspecific inflammatory markers such as c-reactive protein (CRP). Hence, additional biomarkers are needed in order to improve diagnostic specificity and avoid unnecessary further diagnostic testing.

PECAM-1, also denoted as CD31 is a single chain glycopeptid cell surface receptor expressed on platelets [14], endothelial cells [15], macrophages/monocytes [16], neutrophils [17], lymphocytes and bone marrow cells. PECAM-1 is involved in a number of important biological processes including leukocyte transmigration [18], vascular development [19] [20] and thrombus resolution [21]. At the site of venous thrombosis, a soluble subform of PECAM-1 (sPECAM-1) is generated by proteolytic cleavage [21] at the cell surface with consecutive sPECAM-1 plasma level elevation. Studies have already indicated a potential role of sPECAM-1 in venous thrombosis [21, 22]. In contrast to D-Dimer, sPECAM-1 plasma levels seem not to be influenced by pro-inflammatory conditions [21]. The goal of the present study was to determine whether sPECAM-1 improves diagnostic value of D-Dimer in patients with suspected DVT.

## Methods

### Study patients

Patients with clinically suspected DVT of the leg (n = 159) were enrolled at the emergency department of the Kepler University Hospital after providing written informed consent. The study protocol was approved by the local ethics committee. All patients were referred to our hospital because of symptoms or signs suggestive of venous thrombosis. DVT pretest probability was routinely evaluated using the validated Wells Score via questionnaire. This scoring tool considers points for: active cancer (+ 1); calf swelling > 3 cm compared to the asymptomatic leg (+ 1); unilateral superficial veins (+ 1); unilateral pitting edema (+ 1); previous DVT (+ 1), entire leg swelling (+ 1); local tenderness along the distribution of the deep venous system (+ 1); recent cast immobilization or paresis (+ 1); bedridden > 3 days or major surgery in the past 12 weeks (+ 1); alternative diagnosis at least as likely as DVT (− 2). Points were summed into a total score (score range: − 2 to 9). A Wells Score > 2 was defined as high pretest probability; a score ≤ 2 and ≥ 1 as moderate pretest probability and a score < 1 was defined as low pretest probability.

Blood samples for D-Dimer (μg/L), sPECAM-1 (ng/mL) and CRP (mg/dL) determination were collected at baseline. For sPECAM-1 determination blood samples were immediately centrifuged at 4 °C, 2000×*g* for 10 min and stored at − 80 °C until final analysis. Total sPECAM-1 measurements were performed utilizing ELISA (sandwich platinum instant ELISA; Ebioscience, San Diego, USA) according to manufacturer’s instructions.

All patients underwent manual CCUS in order to confirm or exclude the existence of a venous thrombus in the suspected leg. CCUS was performed by an experienced radiologist blinded to the laboratory results. DVT was confirmed by the presence of an incompressible venous segment on CCUS. A D-Dimer > 500 μg/L in the absence of an incompressible venous segment on CCUS was defined as “false positive”. An inflammatory background was defined by elevated levels of CRP > 1 mg/dL (upper limit of normal: < 0.5 mg/dL).

### Statistics

All statistical tests were produced using SPSS version 18.0.2. Categorical data is presented using counts and percentages and for categorical data the Fisher’s Exact Test was used. Continuous measurements (sPECAM-1; D-Dimer; CRP) are presented using arithmetic mean, standard deviation, median, first and third quartile (where appropriate). For the comparison of two independent groups if normality and variance homogeneity was assumed, the two-sample *t* Test was used. If normality and no variance homogeneity was assumed, Welch’s *t* Test was used. If normality was not assumed, the exact Mann–Whitney-U-Test was used. As normality-distribution-test for continuous variables the Kolmogorov–Smirnov-Test with Lilliefors Correction was used at a type-I error-rate of 10%. As test of variance homogeneity for continuous variables the Levene-Test was used at a type-I error-rate of 5%.

Binary logistic regression was used to analyze variables (sPECAM-1/D-Dimer/the combination of these variables) in regard to DVT status: For various cut-points distinct sensitivity/specificity values were calculated, receiver operating curves (ROC) were constructed and ROC-AUC (area under curve) were determined to assess diagnostic value of biomarkers or the combination of biomarkers. The correlation between metric variables was calculated by the Bravais-Pearson-Correlation-Coefficient. Sample size and power calculation was based on preliminary data. A p-value < 0.05 was regarded as statistical significant (Power 90%, two-sided Type-I-error 5%).

## Results

### Study patient baseline characteristics

159 Caucasian patients with suspected DVT of the leg were recruited. Mean age was 61.7 (± 19.2) years and 55.3% (n = 88) were female. The most frequently reported clinical symptoms were pain in the leg (91.3%) and unilateral edema (47.2%). Patients were stratified into low- (20.8%, n = 33), moderate- (22.0%, n = 35) and high-pretest probability (57.2%, n = 91) for DVT using the Wells Score.

DVT was finally diagnosed in 44 out of these 159 patients (27.7%) with the following affected venous segments (proximal thrombus edge): external iliac vein (6.8%), femoral vein (56.8%), popliteal vein (20.5%) and tibial/peroneal veins (15.9%). Concomitant symptomatic pulmonary embolism (assessed via computed tomography pulmonary angiography) was detected in 59.1% (n = 26) of these patients. Mean total Wells Score differed significantly between patients with DVT and patients without DVT (4.1 ± 1.5 versus 1.9 ± 2.0 points; p < 0.001). Characteristics of study patients with and without DVT are shown in Table 1.

**Table 1:** Characteristics and laboratory findings in patients with DVT and without DVT

	DVT (n = 44)	No DVT (n = 115)	p-value
Patient characteristics			
Male (n, %)	22 (50.0)	49 (42.6)	0.476
Age (years ± SD)	67.3 (± 11.5)	59.6 (± 21.0)	0.089
Immobilization/surgery^+^ (n, %)	14 (31.8)	26 (22.6)	0.306
Malignancy (n, %)	11 (25)	2 (1.7)	< 0.001
Thrombophilia (n, %)	1 (2.3)	4 (3.5)	1.000
Previous VTE (n, %)	17 (38.6)	23 (20.0)	0.007
Smokers (n, %)	8 (18.2)	40 (34.8)	0.053
Wells Score			
Total-score (points)	4.1 (± 1.5)	1.9 (± 2.0)	< 0.001
High-pretest probability (%)	93.2	43.5	< 0.001
Moderate-pretest probability (%)	2.3	29.6	< 0.001
Low-pretest probability (%)	4.5	29.9	< 0.001
Biomarkers			
Median sPECAM-1 (ng/mL; 25th/75th perc)	85.9 (76.1/98.0)	68.0 (50.1/86.0)	< 0.001
Median D-Dimer (mg/L; 25th/75th perc)	4.06 (2.35/7.44)	0.73 (0.34/1.46)	< 0.001
Mean CRP (mg/dL; ± SD)	3.2 (± 3.9)	1.8 (± 3.8)	< 0.001

^+^Within the previous 12 weeks

### D-Dimer

A D-Dimer cut-off > 500 μg/L had a diagnostic sensitivity of 97.7% (95% CI 88.0–100%), while patients without DVT (n = 115) had a “false-positive” D-Dimer in 62.6% (n = 72) resulting in a D-Dimer specificity of 37.4% (95% CI 28.6–46.0%). 41.6% (n = 30) of patients with “false positive” D-Dimer showed an inflammatory background with CRP > 1 mg/dL.

D-Dimer levels correlated with total Wells Score [r = 0.41 (95% CI 0.27–0.53; p < 0.001)] and with CRP levels [r = 0.61 (95% CI 0.50–0.71; p < 0.001)]. Patients with “false-positive” D-Dimer had higher levels of CRP compared to patients with D-Dimer ≤ 0.500 μg/L and a normal CCUS (2.6 ± 4.5 mg/dL versus 0.5 ± 0.78 mg/dL; p < 0.001).

### sPECAM-1

**Fig. 1:**
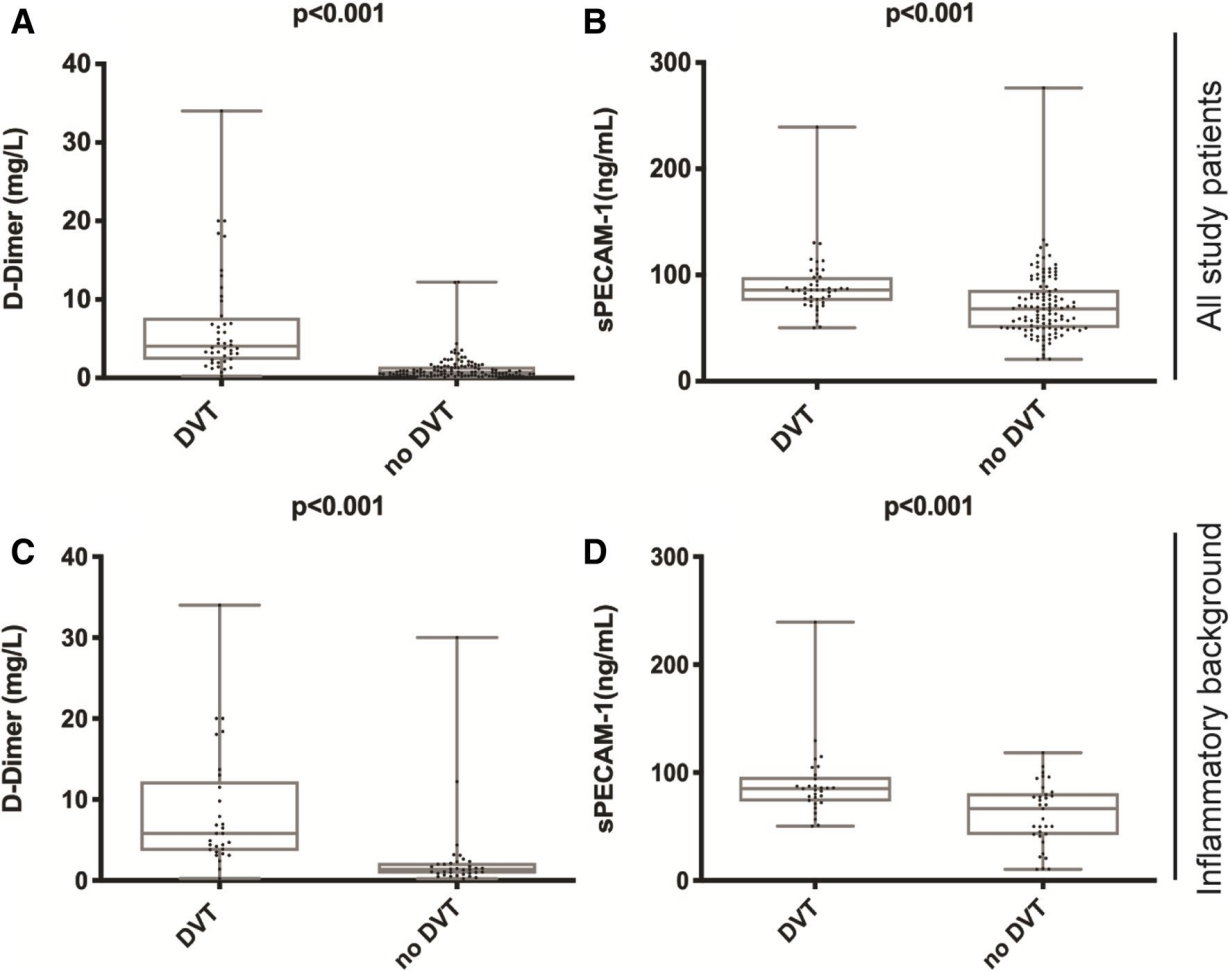
Plasma levels of D-Dimer and sPECAM-1. Boxplots above illustrating D-Dimer levels (mg/L; A) and sPECAM-1 levels (ng/mL; B) in all study patients with DVT vs. without DVT. The two boxplots below show D-Dimer (mg/L; C) and sPECAM-1 levels (ng/mL; D) in patients with an inflammatory background with DVT vs. without DVT

Median sPECAM-1 levels were significantly higher in patients with DVT (85.9 [76.1/98.0] ng/mL) compared to patients without DVT (68.0 [50.1/86.0] ng/mL; p < 0.001; Fig. 1) and levels did not correlate with patient age or CRP levels. Table 2 summarizes diagnostic sensitivity and specificity values of sPECAM-1 at various cut-points. A cut-off > 50.2 ng/mL had a diagnostic sensitivity of 100% (95% CI 92.0–100.0%) and a specificity of 28.7% (95% CI 20.6–37.9%) respectively. Higher levels of sPECAM-1 were more specific for DVT diagnosis. Patients with a low-pretest probability had median sPECAM-1 levels of 69.4 (50.3/82.1) ng/mL compared to 72.4 (52.8/102.0) ng/mL for medium- and 78.1 (58.1/94.3) ng/mL for high-pretest probability.

**Table 2:** Summary of sensitivity/specificity values of sPECAM-1 at various cut-points to distinguish patients with DVT and patients without DVT

sPECAM-1								
Cut-points (ng/mL)	> 20.5	> 50.2	> 71.0	> 80.2	> 94.2	> 105	> 129	> 239
All study patients								
Sensitivity (%)	100	100	86.4	63.6	27.3	13.6	6.8	0
Specificity (%)	0	28.7	58.3	73.9	77.4	87.8	98.3	99.1
Inflammatory background								
Sensitivity (%)	100	100	82.8	62.1	24.1	20.7	6.9	3.4
Specificity (%)	9.1	45.5	57.6	75.8	81.1	93.1	100	100

### Diagnostic value of sPECAM-1 and D-Dimer in all study patients

D-Dimer alone presented a receiver operating curve (ROC) area of 0.905 (p < 0.001) while sPECAM-1 alone showed an AUC of 0.721 (p < 0.001). In the logistic regression analysis, the combination of D-Dimer and sPECAM-1 yielded the best AUC (0.925; p < 0.001) compared to sPECAM-1 alone/D-Dimer alone with a calculated sensitivity of 93.2% (95% CI 81.3–98.6%) and a specificity of 80.9% (95% CI 72.5–87.6%). The combination of D-Dimer (cut-point > 500 μg/L) and sPECAM-1 (cut-point > 50.2 ng/mL) resulted in a reduction of patients with “false-positive” D-Dimer levels from 72 to 49 cases (n = 23; Δ =  − 31.9%). These 23 patients tended to have higher CRP levels compared to the other 49 cases [3.1 (± 4.8) mg/dL vs. 2.3 (± 4.4) mg/dL].

### Patients with an inflammatory background

Laboratory findings of study patients with an inflammatory background are shown in Table 3. 62 out of 159 (38,9%) study patients showed elevated CRP levels > 1 mg/dL. In this subset of patients D-Dimer presented a sensitivity of 96.55% (95% CI 82.2–99.9%) and a specificity of 9.1% (95% CI 1.9–24.3%) with an AUC of 0.870 (p < 0.001) for DVT diagnosis. In the same subset sPECAM-1 displayed a sensitivity of 100% (95% CI 88.6–100.0%) and a specificity of 45.5% (95% CI 28.1–63.5%) (Table 2) with an AUC of 0.846 (p < 0.001) at the cut-off > 50.2 ng/mL. The combination of D-Dimer and sPECAM-1 resulted in a reduction of patients with “false-positive” D-Dimer in this subgroup from 30 to 15 cases (n = 15; Δ =  − 50.0%). In the logistic regression analysis of the subgroup the combination of D-Dimer and sPECAM-1 yielded the best AUC (0.912; p < 0.001) with a calculated sensitivity of 79.3% (95% CI 60.3–92.0%) and a specificity of 87.9% (95% CI 71.8–96.6%).

**Table 3:** Laboratory findings of patients with an inflammatory background with DVT and without DVT

Laboratory parameters	Inflammatory background		
	DVT (n = 29)	No DVT (n = 33)	p-value
Mean leukocyte count (G/L; ± SD)	13.1 (± 2.8)	12.7 (± 2.6)	0.562
Mean platelet count (G/L; ± SD)	187 (± 47)	171 (± 66)	0.282
Median D-Dimer (mg/L; 25th/75th perc)	5.80 (3.80/11.50)	1.34 (0.89/2.00)	< 0.001
Median sPECAM-1 (ng/mL; 25th/75th perc)	85.1 (74.2/94.2)	66.6 (42.9/79.6)	< 0.001
Mean CRP (mg/dL; ± SD)	4.5 (± 4.2)	5.6 (± 5.5)	0.503

## Discussion

In the present study, we evaluated sPECAM-1 as a biomarker for DVT. Two major findings emerge from this study: Firstly, sPECAM-1 levels are significantly higher in patients with DVT as compared to those without DVT. Secondly, sPECAM-1 does not qualify as a diagnostic biomarker alone but improves the diagnostic accuracy of D-Dimer.

D-Dimer is the only routinely used diagnostic biomarker in patients with suspected DVT. However, D-Dimer lacks diagnostic specificity, which limits its implementation as a general screening parameter. A “false-positive” D-Dimer value commonly leads to an avoidable hospital admission and/or unnecessary further imaging. In particular, “false positive” D-Dimer levels in the setting of an inflammatory background is a problem that is frequently encountered. Thus, there is a demand for additional biomarkers to improve biomarker specificity, which could reduce costs and save medical resources.

In our present study, we prospectively recruited 159 patients with clinically suspected DVT and measured D-Dimer and sPECAM-1 levels to evaluate their diagnostic value. DVT was finally diagnosed utilizing CCUS in 44 out of 159 study patients, which is a representative yield in daily practice. All other patients were treated as outpatients with competing diagnoses such as baker cyst rupture, muscular calf pain, arthralgia/arthritis or trauma. In our cohort, D-Dimer showed, as expected, a high sensitivity but a low specificity for DVT diagnosis. False-positive D-Dimer levels mainly occurred in a subset of patients characterized by elevated levels of CRP. This finding indicates that inflammation drives up D-Dimer levels. In contrast to D-Dimer, sPECAM-1 did not correlate with CRP. In fact, sPECAM-1 levels were significantly higher in patients with DVT compared to controls, which is a novel finding in a prospective study. However, sensitivity/specificity calculations revealed, that sPECAM-1 on its own does not qualify for a biomarker. Diagnostic accuracy of sPECAM-1 was inferior to the diagnostic accuracy of D-Dimer. However, sPECAM-1 contributes to improve diagnostic specificity of D-Dimer by reducing the number of “false-positive” D-Dimer by a Δ of −31.9%. Unnecessary and costly imaging could have been avoided in 23 out of 72 patients. The most likely explanation for this finding was that inflammation did not impact on sPECAM-1 levels. Therefore, we analyzed the subset of patients with an inflammatory background, which was defined by CRP > 1 mg/dL. In this subgroup sPECAM-1 (cut-off > 50.2 ng/mL) showed a sensitivity of 100% and a specificity of 45.5% versus a sensitivity of 96.5% and a specificity of 9.1% for D-Dimer (cut-off > 500 μg/L). However, sPECAM-1 still has a low specificity which indicates presence of other confounders apart from inflammation. In 15 out of 30 patients (50%) with an inflammatory background unnecessary imaging could have been avoided.

In daily practice, patients with low or intermediate pretest probability for DVT undergo D-Dimer measurement as the first evaluating step. In these patients, if D-Dimer is elevated (cut-off > 500 μg/L), additional sPECAM-1 determination could be helpful, as levels ≤ 50.2 ng/mL indicate a “false-positive” D-Dimer. Therefore, additional sPECAM-1 measurement in patients with suspected DVT could contribute to save costs and avoid unnecessary imaging.

The present study has its limitations. First, the study has a relatively small sample size as it was powered for the primary research question (difference of sPECAM-1 between patients with DVT and patients without DVT). Second, it is problematic to utilize the same dataset for both sensitivity/specificity determination and statistical model definition.
